# Association of Stressful Life Events with Psychological Problems: A Large-Scale Community-Based Study Using Grouped Outcomes Latent Factor Regression with Latent Predictors

**DOI:** 10.1155/2017/3457103

**Published:** 2017-09-19

**Authors:** Akbar Hassanzadeh, Zahra Heidari, Awat Feizi, Ammar Hassanzadeh Keshteli, Hamidreza Roohafza, Hamid Afshar, Payman Adibi

**Affiliations:** ^1^Department of Biostatistics and Epidemiology, School of Health, Isfahan University of Medical Sciences, Isfahan, Iran; ^2^Psychosomatic Research Center, Isfahan University of Medical Sciences, Isfahan, Iran; ^3^Department of Medicine, University of Alberta, Edmonton, AB, Canada; ^4^Integrative Functional Gastroenterology Research Center, Isfahan University of Medical Sciences, Isfahan, Iran; ^5^Cardiac Rehabilitation Research Center, Cardiovascular Research Institute, Isfahan University of Medical Sciences, Isfahan, Iran; ^6^Department of Internal Medicine, School of Medicine, Isfahan University of Medical Sciences, Isfahan, Iran

## Abstract

**Objective:**

The current study is aimed at investigating the association between stressful life events and psychological problems in a large sample of Iranian adults.

**Method:**

In a cross-sectional large-scale community-based study, 4763 Iranian adults, living in Isfahan, Iran, were investigated. Grouped outcomes latent factor regression on latent predictors was used for modeling the association of psychological problems (depression, anxiety, and psychological distress), measured by Hospital Anxiety and Depression Scale (HADS) and General Health Questionnaire (GHQ-12), as the grouped outcomes, and stressful life events, measured by a self-administered stressful life events (SLEs) questionnaire, as the latent predictors.

**Results:**

The results showed that the personal stressors domain has significant positive association with psychological distress (*β* = 0.19), anxiety (*β* = 0.25), depression (*β* = 0.15), and their collective profile score (*β* = 0.20), with greater associations in females (*β* = 0.28) than in males (*β* = 0.13) (all *P* < 0.001). In addition, in the adjusted models, the regression coefficients for the association of social stressors domain and psychological problems profile score were 0.37, 0.35, and 0.46 in total sample, males, and females, respectively (*P* < 0.001).

**Conclusion:**

Results of our study indicated that different stressors, particularly those socioeconomic related, have an effective impact on psychological problems. It is important to consider the social and cultural background of a population for managing the stressors as an effective approach for preventing and reducing the destructive burden of psychological problems.

## 1. Introduction

Psychological problems, such as depression and anxiety, are among the most common health problems in the world that account for 30% of the global nonfatal disease burden [1, 2]. The burden of psychological disorders is growing with significant negative impacts on health and major social, human rights, and economic consequences in all countries of the world [2]. According to a World Health Organization (WHO) report, millions of people suffer from some forms of these illnesses (approximately 416 million in 1990 to 615 million in 2013) [2, 3]. The prevalence estimates in different surveys as high as 13–30% for depression and 18–31% for anxiety [4–7]. In Iran, the prevalence of these illnesses increased from 11% in 1963 [8] to 34.2% in 2007 [9].

People exposed to stressful life events are more likely to report subsequent psychological problems [10–16]. Stressful life events are described as discrete quantifiable circumstances, such as job conflicts and security, financial problems, social relations, family and personal conflicts, educational concerns, and stressors related to health that can have a severe negative impact on psychological status in which they increase the risk of depression and anxiety [17–20]. Stressors experienced by an individual can affect the body response through activating the sympathetic nervous system and the hypothalamic-pituitary-adrenal (HPA) axis, and such stress reactivity has been associated with increased level of oxidative stress, which seems to accelerate cell aging [21–23]. Accordingly, stressful life events could have a special role in the onset and exacerbation of psychological problems, somatic disorders, and chronic illnesses, such as heart disease, stroke, and type 2 diabetes [17, 22–25].

Studies have been approached the relationship between stressful life events and psychological problems in two ways. One group of studies examined the relationship between a single type of stressful life events (e.g., financial problems, social relations, and family conflicts) and a number of psychological problems or symptoms [19, 20, 26–28]. The second group of studies has relied on composite measures of stressful life events, because a number of these events overlap with each other or can conceptually be combined together [29–33]. Since there are few data about the relationship between stressful life events and psychological problems in the Iranian general population, therefore, the aim of the current study was to address two questions: First, are major psychological problems (i.e., depression, anxiety, and psychological distress) associated with a composite measure of stressful life events? Second, is the composite measure of stressful life events associated with a grouped outcome measured based on the three studied psychological problems? We addressed these questions simultaneously using a comprehensive statistical method, that is, latent factor regression for grouped outcomes with confirmatory latent predictors (stressful life events). In the current study, psychological problems (depression, anxiety, and psychological distress) were considered as the grouped outcomes and stressful life events as latent predictors. Multiple-outcome regression models combine information contained in some related outcome variables; models of this type are more powerful than fitting separate outcome-specific models to detect a significant predictor effect [34, 35]. On the other hand, this modeling approach yields estimates of the predictor effects both at the group level and at the single outcome level. This paper introduces a new model for handling the relationship between grouped outcomes and latent predictors. This model can be considered as an extended version of work of Woodard et al. [34], so that it is a grouped outcomes latent factor regression on latent predictors.

## 2. Materials and Methods 

### 2.1. Study Design and Participants

This cross-sectional study was conducted in the framework of the “Study of the Epidemiology of Psychological, Alimentary Health and Nutrition” (SEPAHAN) project that was performed in two phases on a large sample of Iranian adults [36]. In the first phase, which included different questionnaires on demographic, lifestyle, and nutritional characteristics, among 10087 persons invited to participate, 8691 subjects took part (response rate: 86.16%). Then, a second series of questionnaires, which were designed to collect gastrointestinal and psychological information of participants, were distributed and 6239 questionnaires were completed (response rate: 64.64%). Finally, national identification numbers of participants were used to link the questionnaires from both phases. In the present study, we used data on 4763 participants with completed information. Written informed consent was obtained from all participants. The study was approved by the Bioethics Committee of Isfahan University of Medical Sciences, Isfahan, Iran (projects numbers #189069, #189082, and #189086). More details about the SEPAHAN project are presented elsewhere [36].

### 2.2. Procedures and Assessment of Variables

#### 2.2.1. Assessment of Psychological Variables

*(1) Psychological Distress*. A self-report screening instrument of the 12-item General Health Questionnaire (GHQ-12) was used to assess psychological distress of participants [37]. The scale has a four-point scale (less than usual, no more than usual, rather more than usual, or much more than usual) which asks whether the participant has experienced a particular symptom or behavior recently. A participant's score could be between 0 and 12 points, and a threshold score of 4 or more was used to identify a participant with high distress level. Montazeri et al. validated the questionnaire for the Iranian population with Cronbach's alpha coefficient of 0.87 [37].

*(2) Hospital Anxiety and Depression Scale*. A self-report 14-item screening instrument of the Hospital Anxiety and Depression Scale (HADS) was used for assessing depression and anxiety of participants. The internal consistency of the questionnaire was reported in the Iranian population by a Cronbach's alpha coefficient of 0.78 [38]. It has a 4-point Likert scale ranging from 0 (not present) to 3 (considerable). The anxiety or depression score of respondents could be between 0 and 21 points (0–7: normal; 8–21: mild, moderate, or severe disorder).

#### 2.2.2. Stressful Life Events

Stressors were measured using a valid and self-administered stressful life events (SLEs) questionnaire [39]. It is comprised of 46 items in 11 various dimensions, including home life (measured with addiction, divorce or separation, concern about addiction of a family member, quarrels with spouse, being accused, legal problems, and troubles with children), financial problems (getting in debt, low income, major financial problems, taking on a mortgage, and financial inflation), social relations (social discrimination, major social changes, social insecurity, and concern about your future), personal conflicts (loneliness, lack of social support, cultural alienation, not having an intimate friend, and failure in achieving life goals), job conflicts (quarrel with colleagues/boss, dealing with customers, increased working hours, and improper working place and environment), educational concerns (failure in major examinations, participation in major examinations, high educational expenses, and educational problems of children), job security (job layoff, long-lasting unemployment, concern about job future, high responsibility job, and low salary), loss and separation (death of a close family member, major disease of family members leading to hospitalization, death of parents, spouse, or siblings, and children's separation from family), sexual life (pregnancy, unwanted pregnancy, birth of a child, and sexual relationship problems), daily life (air pollution and traffic, major changes in sleeping and eating habits), and health concerns (mild illness, major physical disease leading to hospitalization). The occurrence of the mentioned stressors was assessed based on a six-point response scale (0 = never; 1 = very mild; 2 = mild; 3 = moderate; 4 = severe; 5 = very severe). The higher score, based on the experience of more events, showed higher stress level. SLE questionnaire has been validated in the Iranian general population [39]. The reliability of the questionnaire was reported by Cronbach's alpha coefficient of 0.92 [39].

#### 2.2.3. Assessment of Other Variables

Self-administered standard questionnaires were used to collect demographic (age, gender, marital status (single/married), education level (≤12 and >12 years of formal schooling), etc.) and lifestyle factors (weight (kg), height (m), and physical activity (inactive and moderately inactive/moderately active and active) based on General Practice Physical Activity Questionnaire (GPPAQ) [40]).

### 2.3. Statistical Analysis

Latent factor regression for grouped outcomes was used for modeling the relationship of stressful life events, as latent predictors, with psychological problems, as the grouped outcomes. In the modeling process, we also adjusted the effect of demographic variables (i.e., age, gender, marital status, and education level) and lifestyle factors (physical activity and body mass index (BMI)).

Quantitative and qualitative variables were expressed as mean ± standard deviation (SD) and number (percentage), respectively. We used independent Student's *t*-test and chi-square tests, where appropriate, to perform between-group comparisons. Spearman rank correlation coefficient was used to investigate the simple associations between dependent and independent variables.

#### 2.3.1. Latent Factor Regression for Grouped Outcomes

Typically, studies aiming to evaluate the effects of predictors on multiple correlated outcomes estimate these effects with fitting regression models to each outcome, separately. However, separate models lack power to detect small but potentially important effects of predictors on multiple correlated outcomes [34, 35]. Unlikely, multiple-outcome regression models share information contained in correlated outcome variables and lead to increased stability of effect estimates and power to detect a significant predictor effect [34, 35]. One the other hand, multiple correlated outcomes may be manifestations of a smaller number of grouped outcomes or domains. In such situations, application of nested domain models can take into account the relationships between multiple-outcome measures nested within domains [34, 35].

As described by Woodard et al., there are two approaches for modeling the relationship of predictor variables with multiple correlated outcomes [34]. One approach, which can be considered as an extended version of the mixed model approach, models the predictors effect on the outcomes directly by using random effects [34]. The second approach is based on continuous latent factors, as manifestation of multiple outcomes. Continuous latent factor models are highly parameterized, but more flexible, while random effect models for multiple outcomes nested in domains are parsimonious, but less flexible [34].

First, according to Woodard et al.'s notations, we briefly describe the random effect model for multiple outcomes [34]. Let *Y*_*ij*_ denote the *j*th (*j* = 1,2,…, *p*) observed response of individual *i* (*i* = 1,2,…, *n*). Note that the *p* outcomes are grouped into *d* domains, which are defined to contain strongly positively correlated outcomes, and each outcome is nested in a single domain. Here, we denoted the scaled and centered covariates by a length-*r* vector *Z*_*i*_. The random effect model can be expressed by using the following regression equation, where it is supposed that the outcome variables and covariates are standardized, and the notation $\begin{array}{c} ind\\ \sim \end{array}$ shows that the random effects are independently distributed:

(1) $$\begin{array}{c} Y_{ij}=\left(\;b_{o,z,j}+b_{D,z,d\left(j\right)}+b_{z}\;\right)Z_{i}+q_{i}+q_{i,d\left(j\right)}+e_{ij}. \end{array}$$

In (1), for *k* = 1,2,…, *d*, **b**_*z*_ is a vector of overall covariate effects, $b_{D,z,k,l}\begin{array}{c} ind\\ \sim \end{array}N(0,\tau_{D,l}^{2})$ is a domain-specific covariate effect for the *l*th covariate, and$\;b_{o,z,j,l}\begin{array}{c} ind\\ \sim \end{array}N(0,\tau_{o,l}^{2})$ is an outcome-specific covariate effect for the *l*th covariate. In addition, $q_{i}\begin{array}{c} ind\\ \sim \end{array}N(0,\tau_{q}^{2})$, $q_{i,k}\begin{array}{c} ind\\ \sim \end{array}N(0,\tau_{q,k}^{2})$, and $e_{ij}\begin{array}{c} ind\\ \sim \end{array}N(0,\sigma_{j}^{2})$ are subject-specific random effect, subject-domain effect, and residual error, respectively. It is assumed that the subject random effect *q*_*i*_ and the subject-domain effect *q*_*i*,*k*_ are sufficient to capture the correlations between the multiple outcomes measured on the same subject even after accounting for covariates and additional correlation between outcomes within a domain, respectively [34].

In contrast with the first approach, in the continuous latent factor model, one or more latent variables are introduced in order to induce correlation between related outcomes, so that the outcomes are viewed as multiple manifestations of the latent variables [34]. The general form of a continuous latent factor model can be expressed by using the following two regression equations:

(2) Yi=α+βo,zZi+Λξi+εiξi=βD,zZi+Bξi+ζi.

Here, the number of latent factors is considered equal to the number of domains and each of the outcomes is assigned to a single domain. In (2), *Y*_*i*_ is the length-*p* vector of outcomes for the *i*th subject, *α* is a length-*p* vector of intercepts, *β*_*o*,*z*_ is a *p* × *r* matrix of regression coefficients, Λ is a *p* × *d* matrix of factor loadings, *ξ*_*i*_ is a length-*d* vector of latent factors, *ε*_*i*_ is a length-*p* vector of independent residuals such that $\varepsilon_{ij}\begin{array}{c} ind\\ \sim \end{array}N(0,\sigma_{j}^{2})$ (cov(*ε*_*i*_) = Σ), *β*_*D*,*z*_ is a *d* × *r* matrix of regression coefficients, *ζ*_*i*_ is a length-*d* vector of residuals such that $\zeta_{i,k}\begin{array}{c} ind\\ \sim \end{array}N(0,\tau_{\zeta ,k}^{2})$ (cov(*ζ*_*i*_) = Ψ), and **B** is a *d* × *d* matrix with zero diagonal elements that induces correlation among the latent factors, and (**I** − **B**) invertible [34].

Woodard et al. used the Bayesian approach, based on Markov chain Monte Carlo, in order to estimate parameters.

#### 2.3.2. Latent Factor Regression for Grouped Outcomes with Latent Predictors

In the previous section, we presented an overview of latent factor regression for grouped outcomes. In the aforementioned models, the independent variables (the length-*r* vector *Z*_*i*_) are measurable or observable. However, in many subject areas such as public health, psychology, and social sciences, there are concepts or constructs that cannot be measured directly by a single measurable variable but they could be measured by a series of observed variables. For example, the various dimensions of stressful life events such as home life, financial problems, and social relations as the predictors of psychological problems cannot be measured directly (latent predictors); they are measured by a series of observable indicators of life event stressors. On the other hand, it is also hypothesized that psychological problems are grouped outcomes of interest. Therefore, in order to directly address our research question (i.e., how psychological problems as the grouped outcomes could be predicted by stressful life events as latent predictors), we need a model for incorporating both types of variables simultaneously. This paper introduces a new model for handling the relationship between grouped outcomes and latent predictors. This model can be considered as an extended version of the continuous latent factor model (see (2)), so that it is a group outcomes regression on latent predictors.

We follow the previous notations; for subject *i* (*i* = 1,2,…, *n*), let *X*_*i*_ = (*X*_*i*1_, *X*_*i*2_,…,*X*_*iq*_)^*T*^ be a *q*-dimensional observed vector with continuous elements used to measure an *m*-dimensional continuous latent variable **η**_*i*_. Then, for any observation vector **X** (*X*_1_, *X*_2_, …, *X*_*q*_), the factor model is as follows:

(3) $$\begin{array}{c} X=\theta \eta +\delta , \end{array}$$

so that

(4) $$\begin{array}{c} f\left(\;X_{i}\;|\;\eta_{i}\;\right)\sim N_{q}\left(\;\left(\begin{array}{c} \theta_{0}\\ 0 \end{array}\right)+\left(\begin{array}{c} \theta \\ I \end{array}\right)\eta_{i},\Psi \;\right), \end{array}$$

where ***θ*** is a *q* × *m* matrix of factor loadings, **η**_*i*_ is a length-*m* vector of latent factors, and *δ*_*i*_ is a length-*q* vector of independent residuals (specific variance) such that $\delta_{ij}\begin{array}{c} ind\\ \sim \end{array}N(0,\psi_{j}^{2})$. Suppose we also have a length-*r* vector *Z*_*i*_ of observable covariates. Therefore, latent factor regression model for grouped outcomes with latent predictors is as follows:

(5) Yi=α+βo,ηηi+βo,zZi+Λξi+εiξi=βD,ηηi+βD,zZi+Bξi+ζi.

Like before, *α* is a length-*p* vector of intercepts, *η*_*i*_ is a length-*m* vector of latent predictors based on (3), *Z*_*i*_ is a length-*r* vector of observable covariates, *β*_*o*,*η*_ and *β*_*o*,*z*_ are *p* × *m* and *p* × *r* matrices of regression coefficients, Λ is a *p* × *d* matrix of factor loadings, *ξ*_*i*_ is a length-*d* vector of latent factors, *ε*_*i*_ is a length-*p* vector of independent residuals such that $\varepsilon_{ij}\begin{array}{c} ind\\ \sim \end{array}N(0,\sigma_{j}^{2}),\;\;\beta_{D,\eta }$ and *β*_*D*,*z*_ are *d* × *m* and *d* × *r* matrices of regression coefficients, *ζ*_*i*_ is a length-*d* vector of residuals such that $\zeta_{i,k}\begin{array}{c} ind\\ \sim \end{array}N(0,\tau_{\zeta ,k}^{2})$, and **B** is a *d* × *d* matrix with zero diagonal elements that induces correlation among the latent factors.

We considered the estimation process of the model parameters via the maximum likelihood method. In the general model formulation, let *ξ*_*i*_ be a length-*d* random vector that represents all latent variables. The measurement part of the model in matrix form is as follows:

(6) $$\begin{array}{c} \left(\begin{array}{c} X_{i}\\ Y_{i} \end{array}\right)=\alpha +\Lambda \xi_{i}+KZ_{i}+\varepsilon_{i}. \end{array}$$

The structure part of the model, which defines a linear structure between the latent variables, in matrix form is as follows:

(7) $$\begin{array}{c} \xi_{i}=\Gamma Z_{i}+B\xi_{i}+\zeta_{i}, \end{array}$$

where **α**, Λ, **K**, Γ, and **B** are parameter matrices. A restriction on **B** is that (**I** − **B**) must be invertible. We consider the dependent data vector into two pieces (**X**_*i*_ and **Y**_*i*_) to represent predictors and outcomes for emphasizing that the model explicitly accommodates predictors measured with error. Finally, the likelihood function of the data **X**_*i*_ and **Y**_*i*_, conditional on covariates **Z**_*i*_, is as follows:

(8) Lθ=∏i=1nfXi,Yi ∣ Zi,θ=∏i=1n∫fXi,Yi ∣ Zi,ξi,θfξi ∣ Zi,θdξi,

where ***θ*** = {**α**, Λ, **K**, Γ, **B**, Σ, Ψ}. Numerical maximization techniques (e.g., EM, Newton-Raphson, and Fisher scoring) can be used to find maximum likelihood estimates.

In the following, we adopted our introduced model and random effect model as a competitor approach and the results of both modeling approaches are presented. Goodness of fit of models was guided through comparing the Akaike Information Criterion (AIC) and the Bayesian Information Criterion (BIC) indices across models. Lower BIC and AIC values indicate better model fitting.

At first, we performed a factor analysis on the 11 stressful life events dimensions, based on principal component extraction approach and orthogonal Varimax rotation procedure. We found two interpretable factors based on the loaded items in each factor; then, in the final model, a confirmatory factor analysis (CFA) was adopted for constructing latent predictors. The following statistics and indices were used for evaluating the goodness of model fitting: the comparative fit index (CFI), the Tucker-Lewis index (TLI), and the root mean square error of approximation (RMSEA). CFI and TLI values range from 0 to 1; values of 0.90 or above indicate acceptable fit. The RMSEA value ranges from 0 to 1, with smaller values of this index indicating better model fit.

Then, the proposed latent factor regression for grouped outcomes with latent predictors (obtained from a confirmatory factor analysis) was fitted among psychological problems (anxiety, depression, and psychological distress) as grouped outcomes and two extracted factors from life events stressors as confirmatory latent predictors. The effects of predictors in crude and adjusted models were evaluated with considering demographic variables (age, gender, marital status, and education level) and lifestyle characteristics (physical activity and BMI) as confounder variables.

## 3. Results

In this study, 4763 subjects with a mean ± SD age of 36.58 ± 8.09 years participated. Demographic characteristics of study participants were as follows: 44.22% male, 81.2% married, and 57.2% university graduated. About 3.5% of individuals were underweight, 36.7% were overweight, and 9.8% were obese. 34.8% of the participants had regular physical activity (moderately active and active) (Table 1). Mean scores of psychological problems and stressful life events are presented in Table 1. Mean ± SD of psychological distress, anxiety, and depression was 2.08 ± 2.74, 3.55 ± 3.72, and 6.15 ± 3.38, respectively. There were significant differences between males and females based on all stressful life events (*P* < 0.01) except sexual life and daily life stressors (Table 1).

### 3.1. Results of Exploratory Factor Analysis (EFA) and Confirmatory Factor Analysis (CFA) on Stressful Life Events

Table 2 provides the factor loadings that resulted from fitting two-factor EFA and CFA on life event stressors. Two factors from stressful life events were extracted using exploratory factor analysis on the 11 stressful life events domains (KMO = 0.86). The two extracted factors were labeled based on the value of factor loadings as “personal stressors domain” and “social stressors domain” (Table 2). The two factors accounted for 17.3% and 25.6%, respectively, of the total variance. Based on the results of CFA, it appears that a two-factor solution provided an appropriate fit to the stressful life event items, because all items loaded significantly on their respective factors. The two-factor CFA showed a good fit both in the total sample (RMSEA = 0.07; CFI = 0.90; TLI = 0.87) and separately in male (RMSEA = 0.07; CFI = 0.91; TLI = 0.88) and female (RMSEA = 0.06; CFI = 0.91; TLI = 0.88) participants.

### 3.2. Correlation between Stressful Life Events and Psychological Problems

The correlation analyses' results for assessing the relationship between scores of stressful life events and scores of psychological problems have been presented in Table 3. All stressful life events correlated significantly with all psychological problems (i.e., psychological distress, anxiety, and depression). Among them, personal conflicts had stronger associations with anxiety (*r* = 0.425, *P* < 0.01) and depression (*r* = 0.412, *P* < 0.01) (Table 3). In addition, there were significant positive relationships between personal stressors domain and psychological problems (*r* = 0.314, 0.406, and 0.346 for psychological distress, anxiety, and depression, resp.; *P* < 0.01). Also, there were significant positive correlations between social stressors domain and psychological problems (*r* = 0.396, 0.466, and 0.416 for psychological distress, anxiety, and depression, resp.; *P* < 0.01).

### 3.3. Association of Stressful Life Events Profiles with Psychological Problems: Results of Latent Factor Regression Model for Grouped Outcomes on Latent Predictors and Random Effect Model

Table 4 reports the information criteria based on fitting random effect and continuous latent factor models for the association of stressful life events profiles with psychological problems in the entire study population and gender subgroups. As shown in the table, AICs and BICs are strongly confirming the goodness of continuous latent factor model.

**Figure 1:**
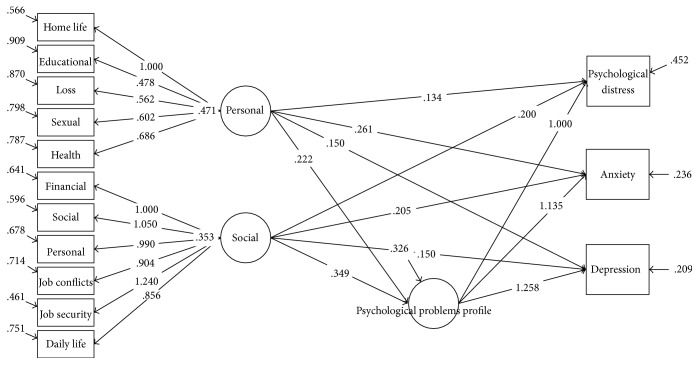
Association of stressful life events profiles scores with psychological problems based on grouped outcomes latent factor regression on latent predictors for the total sample.

**Figure 2:**
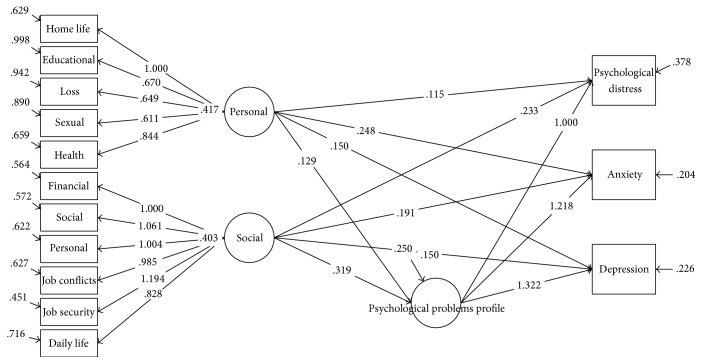
Association of stressful life events profiles scores with psychological problems based on grouped outcomes latent factor regression on latent predictors for males.

**Figure 3:**
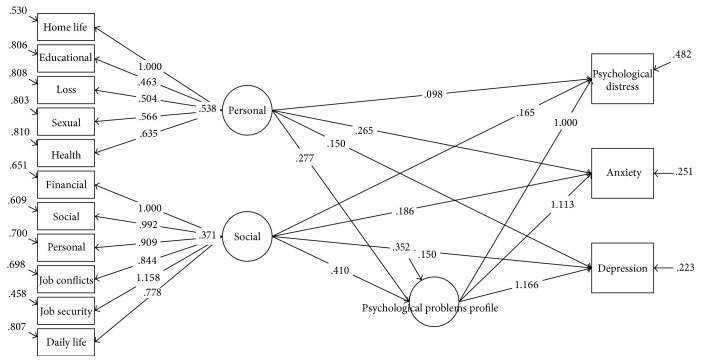
Association of stressful life events profiles scores with psychological problems based on grouped outcomes latent factor regression on latent predictors for females.

Table 5 contains crude and adjusted regression coefficients for the association of stressful life events profiles with psychological problems in the entire study population and gender subgroups using latent factor regression model for grouped outcomes with confirmatory latent predictors and random effect model. The regression coefficients are presented in 2 different models. First, we considered psychological problems (psychological distress, anxiety, and depression) as grouped outcomes and two domains of stressful life events (personal and social domains) as confirmatory latent predictors in the crude model (Figures 1–3).

At the next step, we performed an adjusted model with demographic variables (including age, gender, marital status, and educational level) and lifestyle variables (including BMI and physical activity) as confounder variables. As it was shown in Table 5, the regression coefficients suggest positive associations between both domains of stressful life events with psychological problems profile as well as psychological problems separately in crude and adjusted models based on two modeling approaches. For instance, in the crude and adjusted models, the personal stressors domain had a significantly positive association with psychological problems. The regression coefficients for psychological distress, anxiety, and psychological problem profile score were 0.187, 0.252, and 0.198 (*P* < 0.001), respectively, based on continuous latent factor model. In addition, there is a significant positive relationship between personal stressors domain and psychological problems profile score in both males and females; however, the regression coefficient was greater for females (0.277) (*P* < 0.001) than males (0.129) (*P* < 0.01), based on continuous latent factor model. In the adjusted models, the regression coefficients for the association of social stressors domain and psychological problems profile score were 0.365, 0.353, and 0.462 for the total sample, males, and females, respectively (*P* < 0.001), based on continuous latent factor model. As can be seen, the regression coefficients obtained from random effects modeling approach are higher than the ones obtained from latent factor model, reflecting stronger associations between predictors and dependent variables; however, based on fitting criteria (Table 4), its performance was significantly lower on the one hand, and on the other hand it lacks an important component in linear predictor (i.e., impact of latent predictor on latent dependent variable).

## 4. Discussion and Conclusions

In this cross-sectional population-based study, a comprehensive statistical method (i.e., latent factor regression model for grouped outcomes with confirmatory latent predictors) was introduced to evaluate the association of stressful life events with psychological problems. In the present study, psychological distress, anxiety, and depression were considered as grouped outcomes and two domains of stressful life events (personal and social) as confirmatory latent predictors. Overall, according to the findings of the current study, it was observed that stressful life events directly associated with components of psychological problems and their profile scores, with greater associations in females than in males.

We found a positive association between the personal stressors, including “home life, education, loss and separation, sexual life, and health concerns” with psychological problems and their collective profile scores. In addition, in the current study, there was a positive relationship between the social stressors, including “financial problems, social relations, personal conflicts, job conflicts, job security, and daily life” and psychological disorders and their collective profile scores. Among the stressful life events, personal conflicts had notable association with psychological problems and their profile scores. These findings are in line with some previous studies that documented a significant association between stressful life events and psychological disorders [18, 23, 32, 41–44]. However, it should be noted that the previous studies have focused on the association of stressful life events with psychological disorders such as depression and anxiety separately, but in the current study, we also examined their collective associations through constructing a psychological profile as a latent variable.

In accordance with the present study, Feizi et al.'s study on 4583 people aged 19 and older, living in Isfahan, Iran, showed that family conflicts and social problems are significantly correlated with the levels of perceived stress, which may be related to different Iranian cultural aspects that people are more sensitive to familial and social relationships [23]. Bonde in a meta-analysis showed that psychosocial stressors such as job stressors are related to an elevated risk of subsequent depressive symptoms or major depressive episode [18]. In line with our study, Young and Dietrich [32] and Jensen et al. [33] showed that increases in stressful life events were predictive of both depressive and anxiety symptoms. In Leggett et al.'s longitudinal study on 3,597 adults aged 25 years or older, stressful life events were associated with higher levels of depressive symptoms, and on the other hand experiencing higher levels of stress for a long time was also associated with more depressive symptoms [41]. In accordance with our findings, the study of Mandelli et al. on 415 Italian women aged 18 or more showed that stressful life events especially personal and interpersonal problems and poor social network were positively associated with depressive symptomatology, mood disorder, and suicidal ideation [44]. Park et al. found that stressors, including loss or threatened loss events and loss of a source of self-esteem such as work, finances, or health, were the most common stressful life events preceding the onset of a depressive episode in Asian patients with major depressive disorder [31]. Aktekin et al. among medical students showed that social activities related stressors were associated with psychological problems (anxiety, depression, and GHQ score) [29]. According to the findings of Assari and Lankarani's study, stressful life events predict subsequent risk of developing a major depressive episode; also, they, through a significant gender by stressful life events interaction, concluded a stronger predictive role of stressful life events for subsequent clinical depression for men than for women [24]. In Lim et al.'s study, increased numbers of stressful life events were significantly associated with higher levels of depressive symptomatology among older Chinese adults [30]. The study of Francis et al. also showed that an increased total number of stressful life events were associated with a higher cumulative probability of relapse in anxiety disorder [45]. In Tiet et al.'s study, strong associations were observed between adverse life events and a number of psychiatric disorders such as major depressive disorder [28]. Gjesfjeld et al.'s survey showed that economic stress was associated with increased depressive symptoms through exerting its influence by reducing social support [20]. Tao et al.'s study on Chinese women [46] and also You and Conner's study [47] showed that more severe stressful life events are more strongly associated with depression. In Cutrona et al.'s study on 720 African American women, negative life events (e.g., criminal victimization, natural disaster victimization, serious illness, or injury of a family member) correlated significantly with the onset of major depression [48]. On the other hand, Low et al.'s study indicated that family disruption, interpersonal difficulties stress, and all sources of personal stress were significantly associated with depression symptoms [17]. In some Iranian studies conducted among students, a significant correlation was found between personal and educational stressful life events, such as separation from the family, job concerns, education dissatisfaction, problems with friends, sexual problems, and financial problems, with mental health [49, 50]. Sokratous et al.'s study on 1500 students from Cyprus, 17–40 years old, showed that the students who reported a high number of stressful life events and a severe degree of stress due to these events were more likely to manifest depressive symptoms [51]. Similar findings were reported in Reyes-Rodríguez et al.'s study [52].

In conclusion, the results of the current study indicated that different stressors particularly socioeconomic related ones have effective impacts on psychological problems. The interventions targeted toward promoting financial and social equalities and social skills training have potential benefits in the studied population. In addition, it is important to consider the social and cultural background of a population for managing the stressors as an effective approach for preventing and reducing the destructive burden of psychological problems.

### 4.1. Study Strength and Limitations

It is important to recognize some strengths and limitations of the present study. A major strength of our large population-based study is the application of latent factor regression model for grouped outcomes with confirmatory latent predictors for evaluating the association of stressful life events and psychological disorders. We simultaneously evaluated the association of composite measures of stressful life events with each psychological problem (depression, anxiety, and psychological distress) and a grouped outcome of psychological problems, which lead to more reliable associations. However, due to the cross-sectional design of the study, cause–effect relationships could not be inferred from our findings. It should also be mentioned that all the used information in the present analysis was collected by self-administered questionnaires that might lead to misclassifying the participants. Finally, because SEPAHAN study's participants were working in health centers, thus, generalization of the present findings to the general population in Iran must be done with caution.

## Figures and Tables

**Table 1:** Demographics, lifestyle, psychological characteristics, and stressful life events of the study participants.

Characteristics		Total (*n* = 4763)	Males (*n* = 2106)	Females (*n* = 2657)	*P* value^*∗*^
Demographic characteristics	*Age*	36.58 ± 8.09	38.59 ± 8.61	35.16 ± 7.39	<0.001
	*Marital status*				
	Married	3776 (81.2)	1812 (88.1)	1964 (75.7)	<0.001
	Single	874 (18.8)	245 (11.9)	629 (24.3)	
	*Education level*				
	Undergraduate	1986 (42.8)	1124 (55.0)	862 (33.3)	<0.001
	Graduate	2650 (57.2)	921 (45.0)	1729 (66.7)	

Lifestyle characteristics	*BMI*	25.07 ± 4.64	25.53 ± 4.91	24.72 ± 4.39	
	Underweight	161 (3.5)	45 (2.3)	116 (4.5)	<0.001
	Normal	2282 (50.0)	893 (44.9)	1389 (54.1)	
	Overweight	1672 (36.7)	867 (43.5)	805 (31.3)	
	Obese	445 (9.8)	186 (9.3)	259 (10.1)	
	*Physical activity*				
	Inactive and moderately inactive	2855 (65.2)	1057 (54.9)	1798 (73.4)	<0.001
	Moderately active and active	1522 (34.8)	869 (45.1)	653 (26.6)	

Psychological problems	Psychological distress	2.08 ± 2.74	1.69 ± 2.50	2.38 ± 2.89	<0.001
	Anxiety score	3.55 ± 3.72	2.96 ± 3.44	4.01 ± 3.87	<0.001
	Depression score	6.15 ± 3.38	5.57 ± 3.23	6.60 ± 3.42	<0.001

Stressful life events	Home life	0.65 ± 1.04	0.59 ± 1.02	0.69 ± 1.05	<0.01
	Educational concerns	0.76 ± 1.02	0.81 ± 1.08	0.71 ± 0.97	<0.01
	Loss and separation	0.52 ± 0.73	0.56 ± 0.76	0.49 ± 0.70	<0.01
	Sexual life	0.26 ± 0.54	0.27 ± 0.55	0.26 ± 0.53	0.88
	Health concerns	0.43 ± 0.59	0.37 ± 0.58	0.49 ± 0.60	<0.001
	Financial problems	2.92 ± 1.77	3.15 ± 1.72	2.74 ± 1.79	<0.001
	Social relations	1.75 ± 1.37	1.64 ± 1.39	1.83 ± 1.36	<0.001
	Personal conflicts	1.16 ± 1.28	1.10 ± 1.27	1.21 ± 1.28	<0.01
	Job conflicts	1.73 ± 1.26	1.56 ± 1.26	1.86 ± 1.23	<0.001
	Job security	1.63 ± 1.21	1.69 ± 1.24	1.59 ± 1.19	<0.01
	Daily life	0.59 ± 0.72	0.57 ± 0.71	0.61 ± 0.72	0.07

Values are mean ± SD and number (%). ^*∗*^*P* values were obtained from independent samples *t*-test for continuous data and from Pearson's *χ*^2^ for categorical data.

**Table 2:** Summary results of exploratory and confirmatory factor analysis on stressful life events.

	Total (*n* = 4763)		Males (*n* = 2106)		Females (*n* = 2657)	
	EFA	CFA	EFA	CFA	EFA	CFA
*Personal stressors domain*						
Home life	0.69	0.61	0.65	0.59	0.71	0.64
Educational concerns	0.34	0.42	0.39	0.50	0.36	0.43
Loss and separation	0.58	0.40	0.61	0.40	0.60	0.40
Sexual life	0.61	0.41	0.55	0.38	0.63	0.40
Health concerns	0.57	0.47	0.65	0.52	0.47	0.49

*Social stressors domain*						
Financial problems	0.70	0.61	0.76	0.65	0.63	0.63
Social relations	0.66	0.63	0.64	0.67	0.68	0.61
Personal conflicts	0.53	0.58	0.59	0.62	0.47	0.55
Job conflicts	0.64	0.54	0.66	0.63	0.65	0.52
Job security	0.82	0.72	0.82	0.73	0.79	0.70
Daily life	0.53	0.51	0.53	0.54	0.55	0.48

Values are factor loadings. EFA: exploratory factor analysis; CFA: confirmatory factor analysis.

**Table 3:** Correlation between the scores of stressful life events and the scores of psychological problems.

Stressful life events	Total (*n* = 4763)			Males (*n* = 2106)			Females (*n* = 2657)		
	Psychological distress	Anxiety	Depression	Psychological distress	Anxiety	Depression	Psychological distress	Anxiety	Depression
*Personal stressors domain*	0.314	0.406	0.346	0.295	0.387	0.319	0.328	0.425	0.369
Home life	0.302	0.363	0.313	0.283	0.344	0.265	0.310	0.374	0.340
Educational concerns	0.112	0.158	0.122	0.138	0.165	0.141	0.100	0.168	0.120
Loss and separation	0.106	0.175	0.156	0.094	0.176	0.152	0.125	0.191	0.175
Sexual life	0.170	0.205	0.181	0.154	0.182	0.153	0.184	0.228	0.208
Health concerns	0.259	0.333	0.290	0.239	0.330	0.258	0.254	0.317	0.290

*Social stressors domain*	0.396	0.466	0.416	0.414	0.477	0.406	0.389	0.469	0.432
Financial problems	0.168	0.238	0.213	0.209	0.279	0.226	0.172	0.254	0.247
Social relations	0.365	0.390	0.339	0.380	0.395	0.319	0.342	0.373	0.339
Personal conflicts	0.402	0.425	0.412	0.408	0.422	0.404	0.393	0.424	0.412
Job conflicts	0.216	0.278	0.236	0.207	0.280	0.215	0.196	0.250	0.221
Job security	0.284	0.320	0.273	0.317	0.349	0.299	0.277	0.318	0.271
Daily life	0.245	0.327	0.280	0.254	0.343	0.286	0.234	0.314	0.274

All Spearman rank correlation coefficients are significant at *P* < 0.01.

**Table 4:** The information criteria based on random effect and continuous latent factor models.

	Total (*n* = 4763)		Males (*n* = 2106)		Females (*n* = 2657)	
	AIC	BIC	AIC	BIC	AIC	BIC
Random effect model	166256.770	166532.982	71798.297	72038.769	93967.006	94218.628
Continuous latent factor model	109425.238	109713.926	47217.209	47466.144	62060.700	62322.024

Values are based on crude model (no adjustment was done for confounding variables). AIC: Akaike information criterion; BIC: Bayesian information criterion.

**Table 5:** Crude and adjusted regression coefficients (SE) for the association between stressful life events domains with psychological problems and their profile score based on continuous latent factor and random effect models.

		Total (*n* = 4763)				Males (*n* = 2106)				Females (*n* = 2657)			
		Psychological distress	Anxiety	Depression	Psychological problems profile	Psychological distress	Anxiety	Depression	Psychological problems profile	Psychological distress	Anxiety	Depression	Psychological problems profile
*Personal stressors domain*													
Continuous latent factor model	(1)	0.134 (0.033)^*∗*^	0.261 (0.027)^*∗*^	0.150	0.222 (0.033)^*∗*^	0.115 (0.050)^*∗∗∗*^	0.248 (0.043)^*∗*^	0.150	0.129 (0.045)^*∗∗*^	0.098 (0.043)^*∗∗∗*^	0.265 (0.036)^*∗*^	0.150	0.277 (0.044)^*∗*^
	(2)	0.187 (0.044)^*∗*^	0.252 (0.037)^*∗*^	0.150	0.198 (0.042)^*∗*^	0.135 (0.061)^*∗∗*^	0.240 (0.054)^*∗*^	0.150	0.076 (0.056)	0.138 (0.060)^*∗∗*^	0.252 (0.051)^*∗*^	0.150	0.297 (0.059)^*∗*^
Random effect model	(1)	0.262 (0.015)^*∗*^	0.324 (0.015)^*∗*^	0.271 (0.015)^*∗*^	—	0.202 (0.021)^*∗*^	0.252 (0.021)^*∗*^	0.191 (0.022)^*∗*^	—	0.299 (0.022)^*∗*^	0.370 (0.021)^*∗*^	0.320 (0.020)^*∗*^	—
	(2)	0.228 (0.017)^*∗*^	0.253 (0.016)^*∗*^	0.217 (0.016)^*∗*^	—	0.148 (0.024)^*∗*^	0.185 (0.023)^*∗*^	0.147 (0.025)^*∗*^	—	0.283 (0.024)^*∗*^	0.302 (0.022)^*∗*^	0.262 (0.022)^*∗*^	—

*Social stressors domain*													
Continuous latent factor model	(1)	0.200 (0.034)^*∗*^	0.205 (0.029)^*∗*^	0.150	0.349 (0.035)^*∗*^	0.233 (0.046)^*∗*^	0.191 (0.042)^*∗*^	0.150	0.319 (0.045)^*∗*^	0.165 (0.047)^*∗*^	0.186 (0.041)^*∗*^	0.150	0.410 (0.050)^*∗*^
	(2)	0.208 (0.041)^*∗*^	0.198 (0.034)^*∗*^	0.150	0.365 (0.041)^*∗*^	0.216 (0.055)^*∗*^	0.141 (0.050)^*∗*^	0.150	0.353 (0.053)^*∗*^	0.190 (0.058)^*∗*^	0.164 (0.051)^*∗*^	0.150	0.462 (0.061)^*∗*^
Random effect model	(1)	0.361 (0.015)^*∗*^	0.425 (0.014)^*∗*^	0.415 (0.014)^*∗*^	—	0.381 (0.020)^*∗*^	0.427 (0.020)^*∗*^	0.446 (0.021)^*∗*^	—	0.370 (0.021)^*∗*^	0.452 (0.020)^*∗*^	0.423 (0.019)^*∗*^	—
	(2)	0.393 (0.017)^*∗*^	0.441 (0.016)^*∗*^	0.441 (0.016)^*∗*^	—	0.404 (0.024)^*∗*^	0.415 (0.023)^*∗*^	0.454 (0.025)^*∗*^	—	0.386 (0.023)^*∗*^	0.458 (0.022)^*∗*^	0.437 (0.021)^*∗*^	—

^*∗∗∗*^ *P* < 0.01, ^*∗∗*^*P* < 0.05, and ^*∗*^*P* < 0.1. (1) Crude model: no adjustment was done for confounding variables. (2) Adjusted model: adjustment was done for demographic variables (i.e., age, gender, marital status, and education level) and lifestyle variables (i.e., BMI and physical activity). The gender variable did not adjust for models of males and females.
