# Corrigendum to “Association of Stressful Life Events with Psychological Problems: A Large-Scale Community-Based Study Using Grouped Outcomes Latent Factor Regression with Latent Predictors”

**DOI:** 10.1155/2018/8020962

**Published:** 2018-02-20

**Authors:** Akbar Hassanzadeh, Zahra Heidari, Awat Feizi, Ammar Hassanzadeh Keshteli, Hamidreza Roohafza, Hamid Afshar, Payman Adibi

**Affiliations:** ^1^Department of Biostatistics and Epidemiology, School of Health, Isfahan University of Medical Sciences, Isfahan, Iran; ^2^Psychosomatic Research Center, Isfahan University of Medical Sciences, Isfahan, Iran; ^3^Department of Medicine, University of Alberta, Edmonton, AB, Canada; ^4^Integrative Functional Gastroenterology Research Center, Isfahan University of Medical Sciences, Isfahan, Iran; ^5^Cardiac Rehabilitation Research Center, Cardiovascular Research Institute, Isfahan University of Medical Sciences, Isfahan, Iran; ^6^Department of Internal Medicine, School of Medicine, Isfahan University of Medical Sciences, Isfahan, Iran

In the article titled “Association of Stressful Life Events with Psychological Problems: A Large-Scale Community-Based Study Using Grouped Outcomes Latent Factor Regression with Latent Predictors” [1], the affiliation of the fifth author should be “Cardiac Rehabilitation Research Center, Cardiovascular Research Institute, Isfahan University of Medical Sciences, Isfahan, Iran” only. The corrected author and affiliation lists are shown above.

## References

[1] Hassanzadeh A., Heidari Z., Feizi A. (2017). Association of Stressful Life Events with Psychological Problems: A Large-Scale Community-Based Study Using Grouped Outcomes Latent Factor Regression with Latent Predictors. *Computational and Mathematical Methods in Medicine*.

